# Risk factors for 28-day mortality and pathogen characteristics of septic shock in children: a 10-year retrospective cohort study

**DOI:** 10.3389/fcimb.2026.1780307

**Published:** 2026-05-22

**Authors:** Yanjun Wang, Guizhi Chen, Nana Zhai, Bin Li, Xingxing Feng, Rong Hu, Shufang Xiao

**Affiliations:** 1 Pediatric Intensive Care Unit, Kunming Children’s Hospital, Children’s Hospital Affiliated to Kunming Medical University, Kunming, Yunnan, China; 2 Department of Clinical Laboratory, Kunming Children’s Hospital, Children’s Hospital Affiliated to Kunming Medical University, Kunming, Yunnan, China

**Keywords:** 28-day mortality, children, pathogen characteristics, risk factors, septic shock

## Abstract

**Background:**

Septic shock is a common cause of mortality in critical pediatric patients and pathogen distribution exhibits regional variation. Currently, there is a lack of data regarding septic shock from Southwest China. This study aimed to analyze mortality risk factors between community-acquired septic shock (CASS) and hospital-acquired septic shock (HASS), and changes in pathogen distribution before and after COVID-19 pandemic.

**Methods:**

This retrospective study was conducted at a tertiary pediatric hospital between January 2015 and December 2024 involving children in septic shock. The clinical characteristics, laboratory parameters, pathogen characteristics, and follow-up findings were collected. The patients were divided into CASS and HASS groups. Binary logistic regression analysis was used to identify risk factors for mortality.

**Results:**

A total of 850 patients were included with an average age of 13.0 months (3.7,77.8 months). The 28-d mortality rate was 27.4% and 38.1% in the CASS and HASS groups, respectively. The common infection sites were the respiratory tract (51.2%), digestive tract (27.7%), and central nervous system (7.4%). Multiple organ dysfunction syndrome (MODS) was diagnosed in 68.4% of the patients. The risk factors for mortality in the entire cohort were underlying diseases, lower pediatric critical illness score, bloodstream infection, invasive mechanical ventilation, MODS, renal injury, no vitamin C use, an low platelet count, and an elevated lactate concentration based on multivariate analysis, which were nearly consistent with the CASS group. The pediatric critical illness score and MODS were risk factors in the HASS group. The positive rate of pathogens and blood cultures were 54.1% and 12.6%, respectively. The detection rates for *Escherichia coli* and *Enterococcus* in the post-COVID era showed a downward trend compared to the pre-COVID era, while *Streptococcus pneumoniae*, *Klebsiella pneumoniae*, and *Acinetobacter baumannii* showed an upward trend. In addition, extended-spectrum β-lactamase-producing *E. coli* and methicillin-resistant *Staphylococcus aureus* also increased. Of the HASS patients, 65.1% had hematologic/oncologic diseases, mainly with Gram-negative bacterial infections.

**Conclusion:**

The underlying diseases, pathogens, complications, prognosis, and predictors of mortality varied widely between the CASS and HASS groups.

## Introduction

Sepsis in children is a major cause of morbidity and mortality worldwide, especially in children < 5 years of age (Wang et al., 2014; Fleischmann-Struzek et al., 2018; Schlapbach et al., 2024). The prevalence of sepsis is up to 8% of pediatric intensive care unit (PICU) patients (Weiss et al., 2015). According to the latest international consensus criteria for pediatric sepsis and septic shock (Phoenix Sepsis score), children who met the criteria for sepsis had an in-hospital mortality of 7.1%–28.5% (Schlapbach et al., 2024). Septic shock is a subset of sepsis and is defined as sepsis accompanied by hypotension, the need for vasoactive medications, or evidence of impaired perfusion despite resuscitation with ≥ 40 mL/kg of intravenous fluid boluses, usually resulting in tissue necrosis, multiorgan failure, and death (Singer et al., 2016; Schlapbach et al., 2024). Septic shock among children with sepsis in low-resource settings have significantly higher morbidity (81.3% vs. 53.7%) and hospital mortality rates (33.5% vs. 10.8%) compared to high-resource settings (Schlapbach et al., 2024). In fact, the mortality rate for children with septic shock in China is as high as 34.6% (Wang et al., 2014). Infections are classified as hospital- or community-acquired depending on the site where the infection occurs and have significantly different morbidity and mortality rates (Boeddha et al., 2018; Briassoulis et al., 2021). Identifying the causes of these differences and risk factors for mortality are of vital importance for reducing the risk of death and preventing and treating septic shock.

Research focusing on risk factors associated with mortality and pathogen characteristics in pediatric patients with septic shock is limited, especially in Southwest China. Moreover, changes in pathogen distribution before and after the COVID-19 pandemic have not been established. Murni et al. (2019) reported that 6.4% of patients admitted to a PICU in Indonesia developed nosocomial sepsis with a 41% mortality rate. In another study involving pediatric sepsis in Spain, 22.8% had nosocomial infections, of which 29.0% died (Vila Perez et al., 2014). A study in Peking, China showed that 30.5% of pediatric septic shock cases were nosocomial infections and the 28-d mortality rate was 62.6% and 32.7% in nosocomial and communal septic shock groups, respectively (Su et al., 2022). Given the small sample sizes in the previous studies and the differences in mortality and disease characteristics between nosocomial and community sepsis, data representing large samples are urgently needed to better understand and reconcile these discrepancies.

Understanding the clinical features and distribution of pathogens affecting children with septic shock is essential for treating children with severe infections. In this study we aimed to identify the risk factors for 28-d mortality between community-acquired septic shock (CASS) and hospital-acquired septic shock (HASS), and analyze the changes in pathogen distribution associated with pediatric septic shock before and after the COVID-19 pandemic in a retrospective analysis.

## Materials and methods

### Study design and patients inclusion

**Figure 1:**
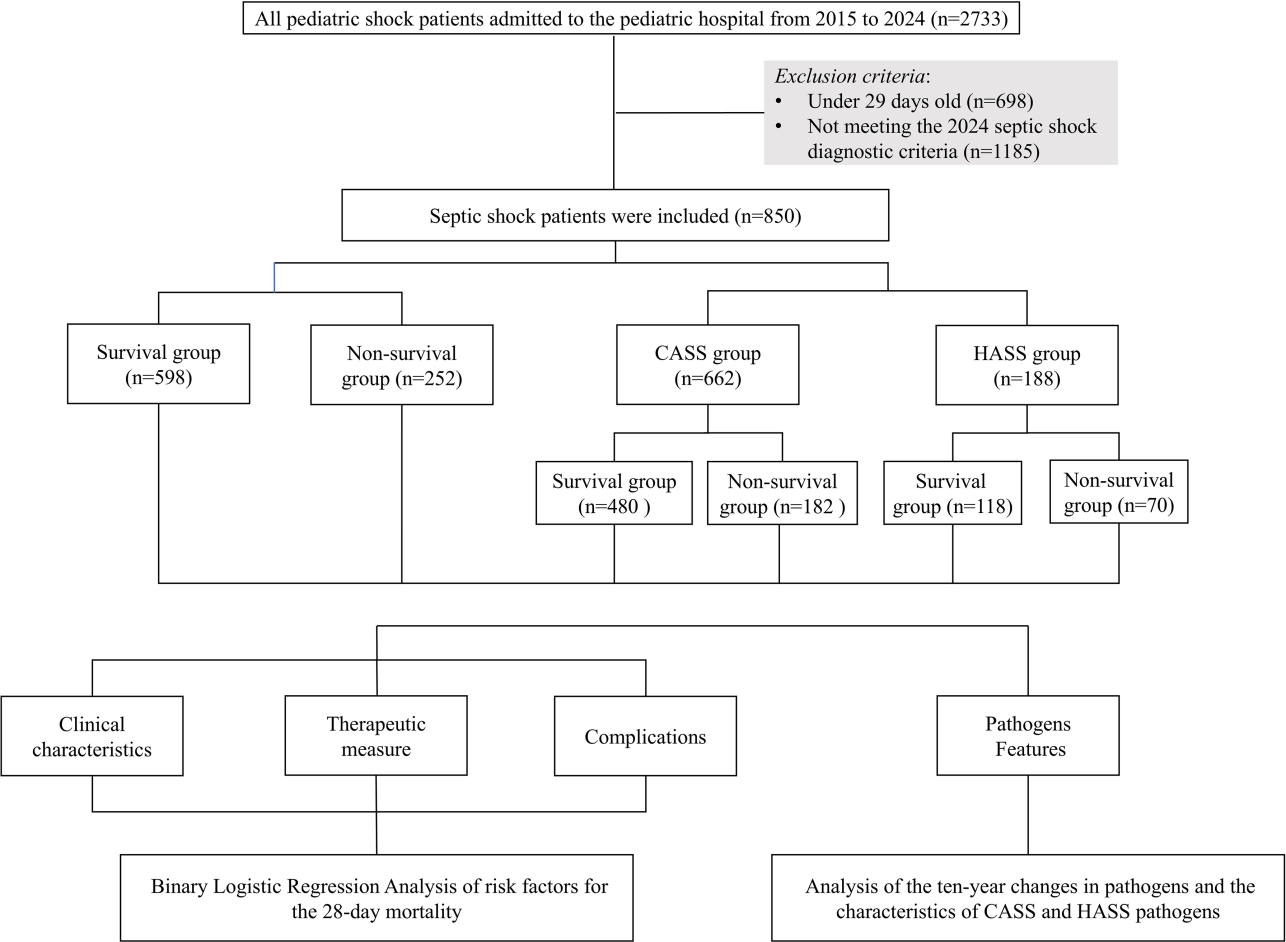
The flowchart for this study.

We conducted a 10-year, single center retrospective cohort study from January 2015 to December 2024. This study included eligible, consecutive children from Kunming Children’s Hospital. A continuous sampling method was used to enhance the representative value of the sample, thereby avoiding the bias caused by random sampling. The inclusion criteria were as follows: 1) 29 d-to-18 years of age; and 2) met the septic shock diagnostic criteria according to the 2024 expert consensus diagnostic standard for children. The exclusion criteria: patients missing > 20% of clinical data. The flowchart for this study is shown in **Figure 1**.

This study adhered to the Declaration of Helsinki and was approved by Kunming Children’s Hospital Ethics Committee (2025-05-105-K01). Because the study was a retrospective analysis of medical records and did not involve any intervention measures, and some patients had been lost to follow-up, the Ethics Committee waived the requirement for informed consent.

### Data collection

Demographic, clinical, diagnostic, etiologic, and therapeutic data from the clinical electronic medical record system, including age, gender, and Pediatric Critical Illness Score (PCIS) items (heart rate, blood pressure, respiratory rate, oxygen partial pressure, pH, serum sodium, potassium, and creatinine, and hemoglobin concentration [The total PCIS ranges from 0–100, with a lower score indicating a more severe condition.]), clinical symptoms, hematologic index (complete blood count, hepatic and renal function tests, and coagulation panel), pathogen testing results, underlying conditions, and nosocomial infection within 24 h of diagnosis, according to previous literature reports and clinical expertise. Co-morbidities, such as respiratory failure, liver function impairment, myocardial damage, coagulation function dysfunction, and acute renal injury were also evaluated. Furthermore, data on the need for continuous renal replacement therapy (CRRT), mechanical ventilation (MV), and vitamin C (100–300 mg/kg), as well as the use of vasoactive agent and anti-pathogen drugs, were also recorded. The 28-d survival after septic shock was obtained through telephone follow-up. The data is stored in a secure electronic database in the form of a variable table.

### Microbiology methods

In this study the collection time of all types of specimens was 48 h before and after the diagnosis of septic shock. The main methods used for pathogen detection included: bacterial and fungal culture of sputum and sterile body fluids; PCR detection of respiratory and intestinal viruses; serum DNA copy number for Epstein-Barr virus (EBV) and cytomegalovirus; and PCR detection or positive IgM serology was adopted for atypical pathogens. After 2020, our hospital introduced next-generation sequencing technology for metagenomics. This test was performed for body fluids with undetected pathogens using traditional detection methods or children with severe infections (accounting for approximately 25.6% of specimens).

### Definition and outcomes measurement

This study applied the 2024 Phoenix Sepsis criteria for the diagnosis of pediatric septic shock, as recommended by the latest expert consensus (Schlapbach et al., 2024). Pediatric septic shock was identified in children with sepsis by at least one point in the cardiovascular component of the Phoenix Sepsis score (i.e., severe hypotension for age, blood lactate > 5 mmol/L, or administration of a vasoactive medication). The Phoenix Sepsis score was calculated using the worst values within 24 h of meeting sepsis criteria based on the following four organ systems: cardiovascular; respiratory; coagulation; and neurologic. Although the study period spans from 2015–2024, the Phoenix Sepsis criteria were applied uniformly to all patients to assure harmonization of historical data. The investigators reviewed raw physiologic and laboratory data from medical records to retrospectively calculate the Phoenix score for patients prior to 2024. The patients were divided into CASS and HASS groups based on the onset of infection within 48 h after admission and progression to septic shock. The CASS group included patients who developed septic shock due to infection within 48 hours of admission or at the time of admission. Specifically, children who had undergone hemodialysis or intravenous chemotherapy within 30 d or who were hospitalized for > 2 d in the past 90 d were classified as patients with healthcare-associated infections and placed in the HASS group (Friedman et al., 2002). The pre-COVID era is defined as the period from January 2015 to December 2019 and the pro-COVID era is defined as the period from January 2020 to December 2024. Cerebral dysfunction is defined as a Glasgow Coma score < 11. The main outcome of this study was the 28-d mortality rate. The secondary outcomes included the mortality rate during hospitalization, the length of stay in the PICU, and the length of hospital stay (LOS).

### Statistical analysis

All statistical analyses were performed using SPSS (version 31.0). The rate of missing values for continuous variables was < 5% and mean imputation was used. The rate of missing values for categorical variables was < 5% and the mode imputation was adopted. Continuous quantitative data following a normal distribution are shown as the mean ± standard deviation. Quantitative data with a non-normal distribution are shown as the median (IQR) values and categorical variables are presented as frequencies (%). Quantitative data for the two groups were analyzed using the Student’s t-test or Wilcoxon rank-sum test. Categorical variables were analyzed using Pearson’s chi-square test or a continuous correction chi-square test. Univariable analyses were used to filter the statistically significant variables for the 28-d mortality rate of children with septic shock and assess multicollinearity. Then, the factors with significant differences (*P* < 0.01) were selected for multivariate logistic regression analysis to identify independent risk factors. The results are reported as the odds ratio (OR) and 95% confidence interval (CI). Finally, a receiver operating characteristic (ROC) curve was drawn to analyze the efficacy of specific risk factors for the 28-d mortality rate. Statistical significance was defined as a *P* < 0.05.

This research report adheres to the STROBE guidelines. The complete STROBE checklist can be found in **Supplementary Material 1**.

## Results

### Clinical features of pediatric patients diagnosed with septic shock

During the study period a total of 850 pediatric patients with septic shock were identified. Girls represented 42.2%(359/850)and the average age was 13.0 months (3.7,77.8 months). Nearly one-half of the patients (46.7% [397/850]) had significant medical and surgical histories, including hematologic/malignant tumor diseases and malnutrition. In this study 662 (77.9%) and 188 (22.1%) patients were in CASS and HASS groups, respectively. The median age was 9.5 months (3.0,40.8 months) and 79.0 months (14.0,136.0 months) in CASS and HASS groups, respectively. A total of 170 (90.4%) patients in the HASS group had underlying diseases, with a predominance of malignant diseases. The HASS group had a higher proportion of blood/malignant tumors and surgical history compared to the CASS group, and a lower proportion of genetic diseases (*P* < 0.05). Children who did not survive were older, had a lower PCIS, and higher proportions of underlying diseases, bloodstream infections, and positive pathogen detection rates compared to children who survived.

Children who did not survive had higher proportions of dyspnea, convulsions and consciousness disorders, lower hemoglobin (Hb) concentrations, platelet (PLT) counts, pH, and albumin (ALB) and fibrinogen (FIB) concentrations, and higher C-reactive protein (CRP), lactate, creatine kinase (CK), creatine kinase isoenzyme (CK-MB), alanine aminotransferase (ALT), and serum creatinine (Scr) concentrations, and higher international normalized ratio (INR), prolonged prothrombin time (PT), and activated partial thromboplastin time (APTT) values compared to children who survived. The HASS group had a lower proportion with convulsions and consciousness disorders, a lower white blood cell (WBC) count, neutrophil count (NC), lymphocyte count (LC), and PLT count, lower Hb, CK, CK-MB, and ALB concentrations, and a lower INR, and higher CRP and total bilirubin (TBIL) concentrations, as well as a prolonged PT compared to the CASS group.

The 28-d in-hospital mortality rate was 29.6% (252/850). The 28-d mortality rate was 27.4% and 38.1% in the CASS and HASS groups, respectively (*P* = 0.005). The characteristics of the septic shock patients are shown in **Table 1**.

**Table 1:** Demographics and clinical features of children with septic shock.

Characteristics	Total (n=850)	Group 1			Group 2		
		Survival (n=598)	Non-survival (n=252)	*P*	CASS (n=662)	HASS (n=188)	*P*
Age, mo	13.0 (3.7,77.8)	10.8 (3.2,67.8)	22.0 (6.5,90.3)	<0.001*	9.5 (3.0,40.8)	79.0 (14.0,136.0)	<0.001*
Female, n (%)	359 (42.2)	239 (39.9)	120 (47.6)	0.039*	278 (41.99)	81 (43.1)	0.789
PCIS, median (IQR)	82 (74,88)	84 (78,88)	76 (68,82)	<0.001*	82 (74,88)	82 (76,88)	0.870
Underlying diseases, n (%)	397 (46.3)	255 (46.2)	142 (56.3)	0.001*	227 (34.3)	170 (90.4)	<0.001*
Malignant disease	167 (19.5)	104 (17.4)	63 (25.5)	0.011*	42 (6.3)	125 (66.5)	<0.001*
History of prior surgery	75 (8.8)	59 (9.9)	16 (6.3)	0.099	47 (7.1)	28 (14.9)	<0.001*
Immunodeficiency disease	13 (1.5)	7 (1.2)	6 (3.0)	0.314	12 (1.8)	1 (0.5)	0.207
Congenital heart disease	36 (4.2)	26 (4.3)	10 (4.0)	0.802	30 (4.5)	6 (3.2)	0.421
Nervous system disease	47 (5.5)	30 (5.0)	17 (6.7)	0.314	41 (6.2)	6 (3.2)	0.112
Genetic diseases	28 (3.3)	16 (2.7)	12 (4.8)	0.120	27 (4.1)	1 (0.5)	0.016*
Malnutrition	62 (7.2)	31 (5.2)	31 (12.3)	<0.001*	48 (7.3)	14 (7.4)	0.927
Infection site, n (%)							
Respiratory tract	544 (63.5)	386 (64.5)	158 (62.7)	0.608	435 (65.7)	109 (58.0)	0.051
Digestive tract	290 (33.8)	213 (35.6)	77 (30.6)	0.155	224 (33.8)	66 (35.1)	0.746
Central nervous system	78 (9.1)	51 (8.5)	27 (10.7)	0.313	64 (9.7)	14 (7.4)	0.352
Bloodstream	133 (15.5)	68 (11.4)	65 (25.8)	<0.001*	79 (11.9)	54 (28.7)	<0.001*
Skin and soft tissue	47 (5.5)	31 (5.2)	16 (6.3)	0.497	30 (4.5)	17 (9.0)	0.017*
Urinary tract	32 (3.7)	30 (5.0)	2 (0.8)	0.003*	28 (4.2)	4 (2.1)	0.181
Unclear site	25 (2.9)	23 (3.8)	2 (0.8)	0.016*	18 (2.7)	7 (3.7)	0.472
Positive pathogen detection, *n* (%)	460 (54.1)	309 (51.7)	151 (59.9)	0.028*	347 (52.4)	113 (60.1)	0.062
Gram-positive bacteria	133 (44.0)	90 (29.1)	43 (28.5)	0.885	99 (28.6)	34 (29.8)	0.456
Gram-negative bacteria	169 (56.0)	108 (35.0)	61 (40.4)	0.255	113 (32.7)	56 (49.1)	0.002*
Symptoms, n (%)							
Dyspnea	235 (27.6)	147 (24.6)	88 (34.9)	0.002*	187 (28.2)	48 (25.5)	0.462
Diarrhea	159 (18.7)	115 (19.3)	44 (17.5)	0.546	125 (18.9)	34 (18.1)	0.805
Convulsions	124 (14.6)	78 (13.0)	46 (18.3)	0.049*	111 (16.8)	13 (6.9)	<0.001*
Consciousness disorder	99 (11.6)	58 (9.7)	41 (16.3)	0.006*	86 (13.0)	13 (6.9)	0.022*
Laboratory inspection, median (IQR)							
WBC (4-10× 10^9/^L)	8.6 (3.9,15.8)	8.7 (4.7,15.6)	8.1 (2.5,16.5)	0.179	9.6 (5.5,16.7)	1.5 (0.3,9.3)	<0.001*
NC (1.8-6.3× 10^9/^L)	5.0 (1.6,9.8)	5.1 (1.8,9.8)	4.4 (0.9,9.6)	0.152	5.8 (2.5,10.7)	0.5 (0.04,6.6)	<0.001*
LC (1.1-3.2× 10^9/^L)	2.2 (0.9,4.0)	2.3 (1.2,3.9)	1.8 (0.7,4.1)	0.103	2.6 (1.4,4.4)	0.6 (0.2,2.1)	<0.001*
Hb (120-140g/L)	105.0 (87.0,121.0)	106 (91.0,120.0)	101.5 (80.3,122.0)	0.019*	108.0 (93.0,123.3)	90.0 (75.0,108.8)	<0.001*
PLT (100-300×10^9^/L)	219.0 (72.0,372.0)	258.5 (112.7,395.0)	117.5 (37.3,279.5)	<0.001*	263.0 (123.8,404.3)	50.5 (21.3,166.3)	<0.001*
CRP (0-10mg/L)	55.9 (7.6,147.9)	51.4 (5.6,135.4)	67.2 (14.0,175.5)	0.014*	41.5 (5.0,130.3)	109.4 (35.4,200.0)	<0.001*
PH (7.35-7.45)	7.35 (7.26,7.41)	7.38 (7.30,7.42)	7.29 (7.15,7.38)	<0.001*	7.35 (7.25,7.40)	7.37 (7.25,7.42)	0.184
BE (-3--**+**3mmol/L)	-8.4 (-13.3,-4.4)	-7.1 (-11.5,-3.9)	-11.7 (-16.6,-6.6)	<0.001*	-8.7 (-13.7,-4.7)	-7.7 (-12.0,-3.7)	0.083
LAC (0.5-2mmol/L)	2.7 (1.7,4.9)	2.3 (1.5,3.8)	4.2 (2.4,8.3)	<0.001*	2.6 (1.6,4.8)	3.1 (1.7,5.5)	0.232
CK (16.5-211.5U/L)	125.0 (55.8,396.2)	109.5 (49.8,281.9)	215.5 (78.3, 826.8)	<0.001*	142.5 (73.0,499.3)	56.0 (25.0,168.3)	<0.001*
CK-MB (15-80U/L)	36.0 (20.0,78.0)	30.0 (19.0,65.0)	66 (26.0,140.8)	<0.001*	42.0 (22.0,98.3)	22.0 (13.0,49.3)	<0.001*
ALT (0-40U/L)	32.0 (18.0,80.5)	29.0 (17.0,62.5)	48.5 (21.0,148.8)	<0.001*	33.2 (18.0,84.8)	29.5 (16.0,76.5)	0.290
ALB (39-54g/L)	32.5 ± 7.4	33.4 ± 7.1	30.5 ± 7.7	<0.001*	33.3 ± 7.6	30.0 ± 6.4	<0.001*
TBIL (3.4-17.7μmol/L)	12.4 (8.0,22.8)	12.0 (8.1,20.9)	13.0 (8.0,26.6)	0.074	11.3 (7.6,19.7)	17.0 (10.0,31.2)	<0.001*
Scr (27-66μmol/L)	31.9 (20.6,61.0)	26.9 (19.4,48.2)	44.7 (26.8,89.1)	<0.001*	31.3 (20.4,63.7)	32.4 (21.2,56.3)	0.798
PT (11-14s)	15.3 (13.6,18.4)	14.7 (13.4,17.2)	16.8 (14.6,21.6)	<0.001*	15.0 (13.5,18.2)	16.0 (14.1,18.5)	0.018*
APTT (20-44s)	41.9 (35.4,50.7)	40.5 (35.0,47.5)	46.3 (37.0,62.1)	<0.001*	41.4 (35.3,50.1)	43.3 (36.4,53.1)	0.097
INR (0.8-1.3)	1.24 (1.09,1.55)	1.20 (1.05,1.43)	1.41 (1.18,1.1.90)	<0.001*	1.23 (1.07,1.54)	1.33 (1.13,1.58)	0.025*
FIB (2-4g/L)	2.64 (1.74,3.85)	2.77 (1.91,4.09)	2.25 (1.34,3.29)	<0.001*	2.66 (1.78,3.95)	2.51 (1.64,3.70)	0.363

*HASS* hospital-acquired septic shock, *CASS* community-acquired septic shock, *PCIS* pediatric critical illness score, *WBC* white blood cell count, *NC* neutrophils count, *LC* lymphocyte count, *Hb* hemoglobin, *PLT* platelet, *CRP* C-reactive protein, *BE* base excess, *LAC* lactate, *CK* creatine kinase, *CK-MB* creatine kinase isoenzyme, *ALT* alanine aminotransferase, *AST* aspartate aminotransferase, *ALB* albumin, *TBIL* total bilirubin, Scr serum creatinine, BUN blood urea nitrogen, *APTT* activated partial thromboplastin time, *PT* prothrombin time, *INR* international normalized ratio, *FIB* fibrinogen, *mo* month, *P<0.05. “History of prior surgery” is defined as any surgical procedure prior to this admission.

### Microbiologic investigations

**Figure 2:**
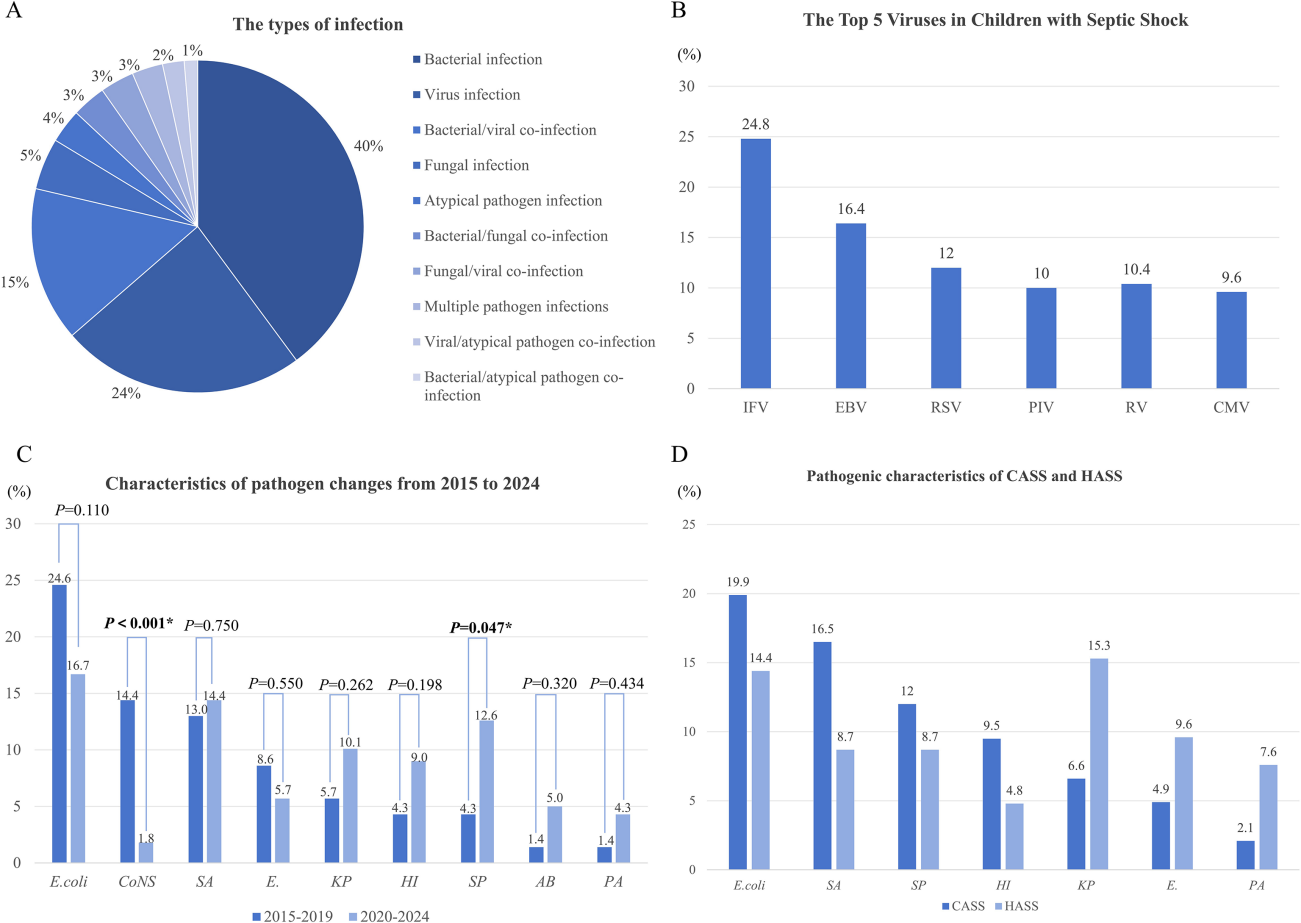
Pathogen distribution in septic shock patients. **(A)** The types of infection. **(B)** The Top 5 Viruses in Children withSeptic Shock. **(C)** Characteristics of pathogen changes from 2015 to 2024. **(D)** Pathogenic characteristics of CASS and HASS.

The most common infection sites in this study were the respiratory tract, digestive tract, bloodstream, and central nervous system. Bloodstream infections were dominant in the children who did not survive and the HASS group (25.8% and 28.7%, respectively; **Table 1**). The pathogen detection rate was 54.1% with Gram-negative bacteria being the predominant type. The positive blood culture rate was 12.6%. The most common types of infection were bacterial infections, virus infections, and bacterial/virus co-infections among the septic shock patients (**Figure 2A**). Of the patients who were pathogen-positive 24% had virus-induced septic shock and 15% had viruses and bacteria co-infections. The detected viruses were mainly influenza A (IFA) virus, EBV, and respiratory syncytial virus [RSV] (**Figure 2B**).

A significant difference in pathogen distribution was observed. Among the 850 children in this study, 250 and 600 were admitted to the hospital during the pre- and pro-COVID eras, respectively. *Escherichia coli*, coagulase-negative *Staphylococci* (*CoNS*), and *Enterococcus* showed a downward trend, while *Staphylococcus aureus* (*SA*), *Klebsiella pneumoniae* (*KP*), and *Streptococcus pneumoniae* (*SP*) showed an upward trend in the post-COVID era compared to the pre-COVID era (*P* < 0.05; **Figure 2C**). The top three pathogenic bacteria in the CASS group were *E. coli*, *SA*, and *SP*, while the top three pathogenic bacteria in the HASS group were *KP*, *E. coli*, and *Enterococcus* (**Figure 2D**). A higher proportion of bloodstream infections and Gram-negative bacteria were noted in the HASS group (*P* < 0.05; **Table 1**).

### Supportive and anti-pathogen therapies

A significant difference was observed in the use of empirical antimicrobial therapy. Most patients in the HASS group were administered ≥ 2 antibiotics (70.2%). In contrast, only 1 antibiotic was administered to most patients in the CASS group (61.2%). Patients in the HASS group were administered higher proportions of carbapenem and glycopeptide antibiotics and antifungal drugs, but a lower proportion received cephalosporin antibiotics compared to patients in the CASS group. The patients who did not survive used a higher proportion of vitamin C than the children who survived (*P* = 0.005; **Table 2**).

**Table 2:** Supportive and antimicrobial therapies in pediatric patients with septic shock.

Characteristics	Total (n=850)	Group 1			Group 2		
		Survival (n=598)	Non-survival (n=252)	*P*	CASS (n=662)	HASS (n=188)	*P*
Respiratory support, n (%)							
NMV	30 (3.5)	24 (4.0)	6 (2.4)	0.239	23 (3.5)	7 (3.7)	0.870
IMV	396 (46.6)	203 (33.9)	193 (76.6)	<0.001*	313 (47.3)	83 (44.1)	0.477
The type of initial antimicrobial drugs							
One antimicrobial drug	464 (54.5)	335 (56.0)	128 (50.8)	0.162	405 (61.2)	58 (30.9)	<0.001*
Two antimicrobial drugs	279 (32.8)	204 (34.1)	75 (29.8)	0.217	201 (30.4)	78 (41.5)	0.004*
More than two antimicrobial drugs	96 (11.3)	48 (8.0)	48 (19.0)	<0.001*	42 (6.3)	54 (28.7)	<0.001*
Antibiotics within 24h of shock onset							
Carbapenems	353 (41.5)	219 (36.6)	134 (53.2)	<0.001*	246 (37.2)	107 (56.9)	<0.001*
Glycopeptides	190 (22.4)	114 (19.1)	76 (30.2)	<0.001*	101 (15.3)	89 (47.3)	<0.001*
Cephalosporins	575 (67.6)	423 (70.7)	152 (60.3)	0.003*	469 (70.8)	106 (56.4)	<0.001*
Antiviral drugs	177 (20.8)	136 (22.7)	41 (16.3)	0.034*	147 (22.2)	30 (16.0)	0.063
Antifungal drugs	124 (14.6)	79 (13.2)	45 (17.9)	0.080	38 (5.7)	86 (45.7)	<0.001*
Vitamin C	513 (60.4)	379 (63.4)	134 (53.2)	0.005*	449 (67.8)	64 (34.0)	<0.001*
Glucocorticoids use	544 (64.0)	371 (62.0)	173 (68.7)	0.067	425 (64.2)	119 (63.3)	0.820
Hemofiltration	30 (3.5)	17 (2.8)	13 (5.2)	0.095	25 (3.8)	5 (2.7)	0.464
Hemoperfusion	13 (1.5)	5 (0.8)	8 (3.2)	0.026*	12 (1.8)	1 (0.5)	0.354
ECMO	3 (0.35)	3 (0.5)	0 (0.0)	0.559	2 (0.3)	1 (0.5)	0.528

*CASS* community-acquired septic shock, *HASS* hospital-acquired septic shock, *NMV* noninvasive mechanical ventilation, *IMV* invasive mechanical ventilation, *ECMO* extracorporeal membrane oxygenation, *P<0.05.

### Complications and outcomes in children with septic shock

A higher proportion of patients who did not survive had complications (*P* < 0.05). The CASS group had more patients with cerebral dysfunction and myocardial damage than the HASS group (*P* < 0.05); no significant differences were noted in the proportion of other complications. The HASS group had a higher mortality rate and a longer LOS compared to the CASS group (*P* < 0.05; **Table 3**).

**Table 3:** The complications and outcomes in pediatric patients with septic shock.

Characteristics	Total (n=850)	Group 1			Group 2		
		Survival (n=598)	Non-survival (n=252)	*P*	CASS (n=662)	HASS (n=188)	*P*
Complications, n (%)							
Respiratory failure	334 (39.3)	172 (28.8)	162 (64.3)	<0.001*	266 (40.2)	68 (36.2)	0.320
Acute renal failure	176 (20.7)	46 (7.7)	130 (51.6)	<0.001*	131 (19.8)	45 (23.9)	0.215
Liver dysfunction	350 (41.2)	210 (35.1)	140 (55.6)	<0.001*	284 (42.9)	66 (35.1)	0.055
Cerebral dysfunction	143 (16.8)	69 (11.5)	74 (29.4)	<0.001*	125 (18.9)	18 (9.6)	0.003*
DIC	16 (1.9)	8 (1.3)	8 (3.2)	0.072	13 (2.0)	3 (1.6)	0.743
Myocardial damage	354 (41.1)	225 (37.6)	129 (51.2)	<0.001*	299 (45.2)	55 (29.3)	<0.001*
MODS	592 (69.6)	355 (59.4)	237 (94.0)	<0.001*	455 (68.7)	137 (72.9)	0.276
Prognosis, n (%)							
Length of hospital stay, d	12.0 (5.0,23.0)	15 (8.0,26.0)	3 (1.0,14.0)	<0.001*	10.0 (4.0,17.0)	24.0 (16.0,35.8)	<0.001*
Length of PICU stay, d	4.0 (1.0,10.0)	5 (2.0,11.0)	2 (1.0,6.0)	<0.001*	5.0 (1.0,10.0)	3.0 (0.0,10.8)	0.013*
28-day mortality, n (%)	252 (29.6)	/	/	/	182 (27.5)	70 (37.2)	0.010*

*CASS* community-acquired septic shock, *HASS* hospital-acquired septic shock, *DIC* disseminated intravascular coagulation, *MODS m*ultiple organ dysfunction syndrome, *PICU* pediatric intensive care unit, *P<0.05,/no data.

### Risk factors predictive of 28-d hospital mortality in pediatric patients with septic shock

The abovementioned variables were included in the univariable logistic regression analysis of the 28-d mortality rate (**Table 4**). Then, the factors with significant differences (*P* < 0.01) were analyzed by multivariate logistic regression. The results excluding the collinear predictors showed that PCIS, combined underlying diseases, bloodstream infections, platelets count, lactate concentration, invasive mechanical ventilation (IMV), vitamin C administration, acute renal injury, and multiple organ dysfunction syndrome (MODS) were risk factors in all children with septic shock (**Table 5**). PCIS, combined underlying diseases, bloodstream infections, platelets count, lactate concentration, IMV, vitamin C administration, and acute renal injury were associated with 28-d mortality in the CASS group. PCIS and MODS were risk factors for 28-d mortality in the HASS group.

**Table 4:** Univariate logistic regression analysis of 28-day mortality in children with septic shock.

Variables	Total		CASS		HASS	
	*P*	OR (95%CI)	*P*	OR (95%CI)	*P*	OR (95%CI)
Age	0.031*	1.003 (1.000-1.006)	<0.001*	1.006 (1.002-1.009)	0.018*	0.994 (0.989-0.999)
PCIS	<0.001*	0.903 (0.885-0.921)	<0.001*	0.898 (0.878-0.918)	<0.001*	0.905 (0.864-0.948)
Positive pathogen detection	0.028*	1.398 (1.037-1.885)	0.134	1.300 (0.922-1.834)	0.130	1.610 (0.869-2.984)
Underlying disease	<0.001*	1.736(1.290-2.337)	<0.001*	1.835 (1.291-2.606)	0.244	0.560 (0.211-1.485)
Bloodstream infection	<0.001*	2.709 (1.855-3.956)	<0.001*	3.185 (1.971-5.147)	0.104	1.705 (0.895-3.246)
CRP	0.013*	1.002 (1.001-1.004)	0.229	1.001(0.999-1.004)	0.071	1.004 (1.000-1.008)
PLT	<0.001*	0.997 (0.996-0.998)	<0.001*	0.997 (0.996-0.998)	0.153	0.998 (0.996-1.001)
ALB	<0.001*	0.947 (0.927-0.966)	<0.001*	0.948 (0.0.926-0.970)	0.062	0.956(0.911-1.002)
TBIL	0.008*	1.002(1.006-1.011)	0.037*	1.036 (1.001-1.011)	0.163	1.007 (0.997-1.017)
APTT	<0.001*	1.030 (1.021-1.039)	<0.001*	1.031 (1.021-1.041)	0.026*	1.023 (1.003-1.044)
INR	<0.001*	2.112 (1.656-2.694)	<0.001*	2.202 (1.683-2.880)	0.055	1.754(0.989-3.113)
LAC	<0.001*	1.213 (1.156-1.273)	<0.001*	1.225 (1.160-1.294)	0.002*	1.185 (1.067-1.316)
IMV	<0.001*	6.365 (4.543-8.917)	<0.001*	7.870 (5.107-12.126)	<0.001*	4.655 (2.468-8.777)
Vitamin C	0.006*	0.656 (0.487-0.884)	0.013*	0.635 (0.445-0.907)	0.957	1.017 (0.545-1.898)
Renal injury	<0.001*	12.787 (8.664-18.872)	<0.001*	9.98 (6.494-15.338)	<0.001*	40.293 (13.350-121.610)
DIC	0.081	2.418 (0.897-6.516)	0.139	2.304 (0.764-6.949)	0.317	3.441 (0.306-38.660)
MODS	<0.001*	10.815 (6.260-18.685)	<0.001*	8.818 (4.874-15.953)	<0.001*	24.145 (5.647-103.245)

*HASS* hospital-acquired septic shock, *CASS* community-acquired septic shock, *PCIS* Pediatric critical illness score, *PLT* platelet, *CRP* C-reactive protein, *TBIL* total bilirubin, *ALB* albumin, *APTT* activated partial thromboplastin time, *INR* international normalized ratio, *LAC* lactic acid, IMV invasive mechanical ventilation, *DIC* disseminated intravascular coagulation, *MODS m*ultiple organ dysfunction syndrome, *CI* confidence interval, *OR* odds ratio, *P<0.05.

**Table 5:** Multivariate regression analysis of risk factors for 28-day mortality.

Variables	Total		CASS		HASS	
	*P*	OR (95%CI)	*P*	OR (95%CI)	*P*	OR (95%CI)
Age	0.927	1.000 (0.995-1.004)	0.639	1.001(0.996-1.007)	0.843	0.999 (0.992-1.006)
PCIS	<0.001*	0.932 (0.909-0.955)	<0.001*	0.937 (0.911-0.964)	0.002*	0.924 (0.879-0.971)
Positive pathogen detection	0.872	1.039(0.650-1.662)	/	/	/	/
Underlying diseases	0.020*	1.685 (1.086-2.616)	0.026*	1.758 (1.069-2.890)	/	/
Bloodstream infection	0.011*	2.262 (1.202-4.256)	0.010*	2.448(1.236-4.849)	/	/
CRP	0.239	0.998(0.995-1.001)	/	/	/	/
PLT	0.039*	0.999(0.997-1.000)	0.456	0.999 (0.998-1.001)	/	/
TBIL	0.683	1.001 (0.995-1.007)	0.769	1.001 (0.994-1.008)	/	/
ALB	0.738	1.005 (0.974-1.038)	0.829	0.996(0.963-1.030)	/	/
APTT	0.147	1.009 (0.997-1.022)	0.108	1.012 (0.997-1.027)	0.798	1.003 (0.981-1.025)
INR	0.793	0.963 (0.727-1.276)	0.772	0.955(0.699-1.305)	/	/
LAC	<0.001*	1.125 (1.060-1.194)	<0.001*	1.128(1.055-1.206)	0.082	1.109 (0.987-1.245)
IMV	<0.001*	3.473 (2.152-5.605)	<0.001*	2.955 (1.665-5.243)	0.192	1.777 (0.749-4.217)
Vitamin C	<0.001*	0.314 (0.195-0.505)	<0.001*	0.312 (0.179-0.545)	/	/
Renal injury	<0.001*	8.158 (4.890-13.611)	<0.001*	6.523 (3.671-11.591)	/	/
MODS	0.003*	3.060 (1.465-6.392)	0.070	2.167 (0.938-5.005)	0.032*	5.878 (1.168-29.576)

*CASS* community-acquired septic shock, *HASS* hospital-acquired septic shock, *PCIS* Pediatric critical illness score, *CRP* C-Reactive protein, *PLT* platelet, *TBIL* total bilirubin, *ALB* albumin, *APTT* activated partial thromboplastin time, *INR* international normalized ratio, *LAC* lactic acid, IMV invasive mechanical ventilation, *MODS* multiple organ dysfunction syndrome, *CI* confidence interval, *OR* odds ratio, *P<0.05,/no data.

### Efficacy of specific risk factor analysis

**Figure 3:**
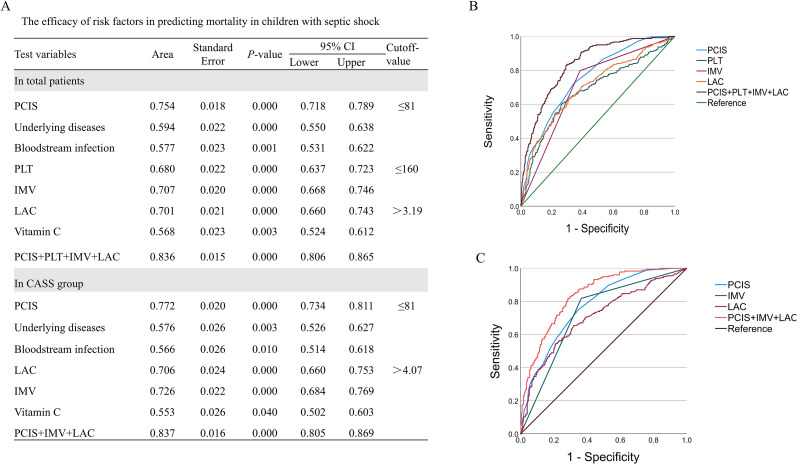
Area under the ROC curve analysis of risk factors to distinguish between the survival and non-survival groups. **(A)** The efficacy of risk factors in all patients. **(B)** Area under the ROC curve analysis of risk factors in all patients. **(C)** Area under the ROC curve analysis of risk factors in CASS patients.

Received operating characteristic (ROC) curve analysis was performed to assess the efficacy of risk factors. IMV, platelets count, lactate concentration, and PCIS had predictive value for poor prognosis in all patients (area under the ROC curve [AUC] = 0.836, sensitivity = 83.5%, specificity = 70.0%) **Figures 3A, B**). PCIS, IMV and lactate concentration had predictive value for mortality (AUC = 0.837) with a sensitivity of 83.0% and a specificity of 70.3% in the CASS group (**Figures 3A, C**).

## Discussion

This study involved a retrospective analysis of pediatric patients with septic shock at a children’s hospital in the southwest region of China from 2015–2024. The main findings were as follows: (1) The pathogen spectrum varied in different areas and changed after COVID-19. (2) The mortality rate of septic shock was 29.6%, which was similar to recent reports in China (Su et al., 2022). (3) There were differences between the CASS and HASS groups. (4) The use of vitamin C was associated with a reduced mortality rate but this association should be interpreted with caution. (5) Lactate levels, the PCIS, and IMV were independent risk factors for mortality.

The pathogen positivity rate in children with severe sepsis in the PICU ranges from 50%–70% (Boeddha et al., 2018; Xiao et al., 2019). The pathogen positivity rate in children with septic shock in this study was 54.2% and the positive blood culture rate was 12.6%. Foreign reports indicated that Gram-positive bacteria are more frequently detected in children with severe sepsis (Thavamani et al., 2020). However, Gram-negative bacteria are more common in China (Xiao et al., 2019). In this study Gram-negative bacteria accounted for 56% of all the detected bacteria. This regional data disparity might be related to the differences in the study population. First, this study excluded cases of neonatal sepsis, which were mainly caused by Gram-positive bacteria. Second, Gram-negative bacteria can induce higher levels of cytokines and are more likely to cause activation and damage of vascular endothelial cells, thereby leading to secondary circulatory dysfunction (Surbatovic et al., 2015). All the participants in this study had septic shock. The bacterial resistance patterns in the southwestern region of China have regional characteristics. The conclusions of this study may be influenced by the local microbial ecology and resistance spectra. The success rate of empirical anti-infective treatment may vary in other regions with different resistance patterns, thereby affecting the overall mortality rate and estimation of the effect of treatment measures.

Clinicians generally do not pay sufficient attention to virus-related sepsis (Kocak Tufan et al., 2021). It has been reported that the positive viral infection rate in children with severe sepsis is between 6.1% and 21% (Xiao et al., 2019), while there are relatively few reports on the proportion of viruses in children with septic shock. Previous studies have shown that respiratory viruses and EBV, among others, often cause organ dysfunction, including circulatory disorders, by directly inducing the release of large amounts of cytokines or by subsequently causing secondary infections by other pathogens (Gu et al., 2020). This study showed that among the detected pathogens, viruses accounted for 24% with IFA, EBV, and RSV the most common.

The mortality rate of children with severe sepsis varies greatly among different reports worldwide (Wang et al., 2014; Weiss et al., 2015; de Souza and MaChado, 2019). With the continuous promotion of international sepsis guidelines, the mortality rate in some regions with advanced medical care has decreased from 40%–60% to ≤ 10% but in regions with underdeveloped medical care, the mortality rate still fluctuates between 20% and 50% (Rech et al., 2022). A multi-center prospective cross-sectional study in China (2018–2019) reported a mortality rate of 18.3% (Wang et al., 2023), while the mortality rate in this retrospective study was 29.6%. Both results indicate that with improvements in medical and health conditions in China, the early identification, diagnosis, and treatment of pediatric septic shock have improved and the mortality rate has significantly decreased.

Several studies (Dabar et al., 2015; Su et al., 2022) have reported differences between CASS and HASS patients, but there is a lack of data involving pediatric patients from Southwest China. In this study the pediatric patients in the CASS and HASS groups exhibited several differences in underlying diseases, mortality rates, risk factors for poor prognosis, and pathogen distribution. The HASS group was older and had a higher mortality rate due to immune deficiency or neutropenia caused by malignant diseases compared to the CASS group, which is consistent with published data (Hall et al., 2016; Pana et al., 2017; Battaglia and Hale, 2019). This study was conducted in a tertiary grade A hospital in the southwest region of China, which is a regional oncology treatment center for children. Therefore, the proportion of patients with malignant hematologic diseases/tumors in the HASS group was relatively high (66.5%). This proportion was much higher than most general pediatric hospitals (Liu et al., 2024). HASS patients tend to present with bloodstream infections due to toxic effects of chemotherapy on the gastrointestinal mucosa, which results in enterogenic sepsis (Baker and Satlin, 2016; Westphal et al., 2019). The predominant infection site in the CASS group was the respiratory tract, which is consistent with the findings of a previous study (Westphal et al., 2019). Previous studies showed significant differences in microbiologic infection profiles between HASS and CASS patients (Matta et al., 2018; Droz et al., 2019). HASS patients are at high risk for Gram-negative bacteremia, which was confirmed by the current study.

There was a difference in the 28-d mortality rates in the HASS and CASS groups. The mortality rates were 27.4% and 38.1% in the CASS and HASS groups, respectively, which were lower than previous small-sample and single-center studies (Dabar et al., 2015; Su et al., 2022). The current study included a larger number of cases and the data were more accurate. A significant discrepancy in pathogen distribution was detected between the pre- and post-COVID eras, as well as the HASS and CASS groups. *E. coil*, *CoNS*, and *Enterococcus* exhibited a downward trend, while *SA*, *KP*, and *SP* exhibited an upward trend in the post-COVID era compared to the pre-COVID era. This is the first report on the changes in pathogens among children with septic shock before and after the COVID epidemic, which might have a significant role in guiding diagnosis and treatment. During the pandemic, possible monitoring biases, such as intensified testing for respiratory pathogens, may lead to an artificially elevated virus detection rate. This bias may overestimate the impact of COVID-19 on the pathogen spectrum. In the CASS group, the top three pathogenic bacteria are *E. coli*, *SA*, and *SP*, while in the HASS group, the bacteria are *KP*, *E. coli*, and *Enterococcus.* The PICU is a high-risk area for the detection of drug-resistant bacteria. Gram-negative bacteria are the most commonly resistant and can lead to a higher mortality rate (Rhee et al., 2020). A pediatric study in China reported an overall resistance rate of 60.1% for Gram-negative bloodstream infections (Dong et al., 2017). The high drug resistance rate of community infections requires the attention of medical staff and society and is of great significance to enhance the monitoring of pathogens and drug resistance, reduce the irrational use of anti-infective drugs, and explore more scientific and effective prevention strategies.

The 28-d mortality rate risk factors differed between the CASS and HASS groups. The multivariable logistic regression analysis showed that a lower PCIS, underlying diseases, bloodstream infections, a low platelets count, an elevated lactate concentration, IMV, acute renal injury, and MODS were risk factors for 28-d mortality, while using vitamin C was associated with lower 28-d mortality in all patients. Risk factors in the CASS group were consistent with the entire cohort except MODS. The risk for 28-d mortality increased in HASS patients with a lower PCIS or MODS. A lower PCIS was related to mortality, which is consistent with a previous study (Zhang et al., 2022). A previous study showed that bloodstream infections acquired in the ICU were associated with a high mortality rate, which is consistent with our findings (Adrie et al., 2017). Previous research involving septic shock patients ranges from children-to-adults and showed that mortality could be predicted by an elevated lactate concentration, especially among patients with community-acquired infections (Ryoo et al., 2018; Alam and Gupta, 2021; Choi et al., 2021), which is consistent with the findings herein. As previously reported (de Montmollin et al., 2016; Menon et al., 2022), IMV is associated with a poor prognosis. Indeed, respiratory failure might be a predictor of mortality, which has been verified in a previous study (Keim et al., 2023). Patients with acute renal injury are at high risk for poor outcomes (White et al., 2023; Qiao et al., 2024), which was also demonstrated in the current study. Renal tubular epithelial cells are the most metabolically active cells in the kidney and are sensitive to sepsis-related damage (Qiao et al., 2024). MODS, a severe clinical manifestation, has been verified in previous studies (Weiss et al., 2017; Tang et al., 2024) to be a risk factor for poor outcome in patients with sepsis. Of septic shock patients in the current study, 69.6% had MODS and 94.0% of patients in the non-survival group had MODS.

### Limitations

This study had several limitations. First, this was a single-center study. All the data were sourced from Kunming Children’s Hospital. Therefore, the sample may not fully represent a broader population and there might be an admission rate bias, which limits the extrapolation of the results. Second, this study had some limitations with respect to unified diagnostic criteria. Although we conducted a retrospective reclassification of all historical cases in strict accordance with the 2024 Phoenix standards, some clinical variables used for Phoenix scoring might not have been fully recorded in the early medical records given the retrospective design, which could lead to classification bias for the early cases. However, by focusing on extracting objective hard endpoint indicators, such as the use of vasoactive drugs and lactate levels, we endeavored to minimize this potential bias as much as possible. Third, not all of the participants received targeted sequencing of multiple pathogens and a considerable number of participants underwent pathogen-specific PCR detection and sputum/blood culturing. Therefore, there was a bias in the methods for pathogen detection. Fourth, because some patients were transported to our hospital after septic shock developed, clinical data at the time of diagnosis could not be obtained. Fifth, some of the treatment measures observed in this study, such as the use of vitamin C, reflect the clinical protocols and physician preferences of this center rather than global standard practices. The association between vitamin C and mortality reported herein may be partially confounded by the unique supportive treatment strategies specific to our center. Therefore, this finding at a single-center level may not be reproducible in centers with different treatment cultures. Finally, as a retrospective study, we cannot rule out the possibility of selection bias due to incomplete medical record information or the subjective judgment of the physicians. In the future, large-scale, multi-center, and prospective studies need to be conducted and the follow-up rate should be maximized as much as possible to further validate the conclusions of this study and reduce the influence of selection bias.

## Conclusions

Higher mortality was observed in children with septic shock who presented with a lower PCIS, underlying diseases, bloodstream infections, a low platelet, an elevated lactate concentration, IMV, no vitamin C, acute renal injury, and MODS. The predictors of administration 28-d mortality differed between HASS and CASS pediatric patients. The underlying diseases, pathogens, complications, prognosis, and mortality rates varied widely between the two groups.

## Data Availability

The original contributions presented in the study are included in the article/**Supplementary Material**. Further inquiries can be directed to the corresponding author.
