# Supplementary material for: Risk factors for 28-day mortality and pathogen characteristics of septic shock in children: a 10-year retrospective cohort study
Source: Front Cell Infect Microbiol. 2026 May 22;16:1780307. doi: 10.3389/fcimb.2026.1780307 (PMC13236943; doi:10.3389/fcimb.2026.1780307)
Supplement: Supplementary file 1 [file DataSheet1.docx]

|  | **Item**  **No** | **Recommendation** | Manuscript page |
| --- | --- | --- | --- |
| **Title** **and** **abstract** | 1 | (a) Indicate the study’s design with a commonly used term in the title or the abstract | Page 2, line 2 |
|  |  | (*b*) Provide in the abstract an informative and balanced summary of what was done and what was found | Page 2, line 6-9 |
| **Introduction** | | |  |
| Background/rationale | 2 | Explain the scientific background and rationale for the investigation being reported | Page 4, line 8-9 |
| Objectives | 3 | State specific objectives, including any prespecified hypotheses | Page 4, line 9-13 |
| **Methods** | | |  |
| Study design | 4 | Present key elements of study design early in the paper | Page 4, line 16-20 |
| Setting | 5 | Describe the setting, locations, and relevant dates, including periods of recruitment, exposure, follow-up, and data collection | Page 4, line 16-20 |
| Participants | 6 | (*a*) *Cohort* *study*—Give the eligibility criteria, and the sources and methods of selection of participants. Describe methods of follow-up  *Case-control* *study*—Give the eligibility criteria, and the sources and methods of case ascertainment and control selection. Give the rationale for the choice of cases and controls  *Cross-sectional* *study*—Give the eligibility criteria, and the sources and methods of selection of participants | Page 4, line 20-24 |
|  |  | (*b*) *Cohort* *study*—For matched studies, give matching criteria and number of exposed and unexposed  *Case-control* *study*—For matched studies, give matching criteria and the number of controls per case | Page 4, line 20-24 |
| Variables | 7 | Clearly define all outcomes, exposures, predictors, potential confounders, and effect modifiers. Give diagnostic criteria, if applicable | Page 5-6 |
| Data sources/  measurement | 8* | For each variable of interest, give sources of data and details of methods of  assessment (measurement). Describe comparability of assessment methods if there is more than one group | Yes |
| Bias | 9 | Describe any efforts to address potential sources of bias | Page 6, line 19-30 |
| Study size | 10 | Explain how the study size was arrived at | N/A |
| Quantitative variables | 11 | Explain how quantitative variables were handled in the analyses. If applicable, describe which groupings were chosen and why | Page 6, line24-27 |
| Statistical methods | 12 | (*a*) Describe all statistical methods, including those used to control for confounding | Page 6 |
|  |  | (*b*) Describe any methods used to examine subgroups and interactions | Page 6, line 7-18 |
|  |  | (*c*) Explain how missing data were addressed | Page 4, line 23-24 |
|  |  | (*d*) *Cohort* *study*—If applicable, explain how loss to follow-up was addressed  *Case-control* *study*—If applicable, explain how matching of cases and controls was addressed  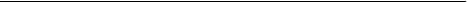*Cross-sectional* *study*—If applicable, describe analytical methods taking account of sampling strategy | N/A |
|  |  | (*e*) Describe any sensitivity analyses | N/A |

STROBE Statement—checklist of items that should be included in reports of observational studies

Continued on next page

| **Results** |  |  |  |
| --- | --- | --- | --- |
| Participants | 13* | (a) Report numbers of individuals at each stage of study—eg numbers potentially eligible, examined for eligibility, confirmed eligible, included in the study, completing follow-up, and analysed | Page 7, line 10-12 |
|  |  | (b) Give reasons for non-participation at each stage | N/A |
|  |  | (c) Consider use of a flow diagram | Figure 1 |
| Descriptive  data | 14* | (a) Give characteristics of study participants (eg demographic, clinical, social) and information on exposures and potential confounders | Page 6, line 10-22 |
|  |  | (b) Indicate number of participants with missing data for each variable of interest | N/A |
|  |  | (c) *Cohort* *study*—Summarise follow-up time (eg, average and total amount) | 28-day mortality |
| Outcome data | 15* | *Cohort* *study*—Report numbers of outcome events or summary measures over time | N/A |
|  |  | *Case-control* *study—*Report numbers in each exposure category, or summary measures of exposure | N/A |
|  |  | *Cross-sectional* *study—*Report numbers of outcome events or summary measures | N/A |
| Main results | 16 | (*a*) Give unadjusted estimates and, if applicable, confounder-adjusted estimates and their precision (eg, 95% confidence interval). Make clear which confounders were adjusted for and why they were included | Page 9, line 14-26 |
|  |  | (*b*) Report category boundaries when continuous variables were categorized | Page 6, line 10-22 |
|  |  | (*c*) If relevant, consider translating estimates of relative risk into absolute risk for a meaningful time period | N/A |
| Other analyses | 17 | Report other analyses done—eg analyses of subgroups and interactions, and sensitivity analyses | N/A |
| **Discussion** |  |  |  |
| Key results | 18 | Summarise key results with reference to study objectives | Page 10, line 5-12 |
| Limitations | 19 | Discuss limitations of the study, taking into account sources of potential bias or imprecision. Discuss both direction and magnitude of any potential bias | Page 6, line 10-22 |
| Interpretation | 20 | Give a cautious overall interpretation of results considering objectives, limitations, multiplicity of analyses, results from similar studies, and other relevant evidence | Page 13, line 21- Page 14, line 18 |
| Generalisability | 21 | Discuss the generalisability (external validity) of the study results | Page 14, line 13-18 |
| **Other** **information** | | | |
| Funding | 22 | Give the source of funding and the role of the funders for the present study and, if applicable, for the original study on which the present article is based | Page 14, line 26 |
